# Strategies and challenges associated with recruiting retirement village communities and residents into a group exercise intervention

**DOI:** 10.1186/s12874-018-0633-4

**Published:** 2018-12-20

**Authors:** Rachel L. Duckham, Jamie L. Tait, Caryl A. Nowson, Kerrie M. Sanders, Dennis R. Taaffe, Keith D. Hill, Robin M. Daly

**Affiliations:** 1 Institute for Physical Activity and Nutrition, Deakin University, 221 Burwood Highway, Burwood, VIC 3125 Australia; 2 Australian Institute for Musculoskeletal Sciences (AIMSS), St. Albans, Melbourne, Australia; 3 Department of Medicine, Western Health, Department of Medicine, University of Melbourne, Sunshine Hospital, St Albans, Victoria Australia; 4 Australian Institute for Musculoskeletal Sciences (AIMSS), University of Melbourne and Western Health, Melbourne, St. Albans Australia; 5 Exercise Medicine Research Institute, Edith Cowan University, Perth, Western Australia Australia; 6 School of Medical and Health Sciences, Edith Cowan University, Perth, Western Australia Australia; 7 School of Human Movement and Nutrition Sciences, University of Queensland, Brisbane, Queensland Australia; 8 School of Physiotherapy and Exercise Science, Curtin University, Perth, Western Australia Australia

**Keywords:** Recruitment strategies, Retirement communities, Dual-task training, Falls, Older adults, Clustered randomised controlled trial

## Abstract

**Background:**

Randomized controlled trials (RCTs) provide the highest level of scientific evidence, but successful participant recruitment is critical to ensure the external and internal validity of results. This study describes the strategies associated with recruiting older adults at increased falls risk residing in retirement villages into an 18-month cluster RCT designed to evaluate the effects of a dual-task exercise program on falls and physical and cognitive function.

**Methods:**

Recruitment of adults aged ≥65 at increased falls risk residing within retirement villages (size 60–350 residents) was initially designed to occur over 12 months using two distinct cohorts (C). Recruitment occurred via a three-stage approach that included liaising with: 1) village operators, 2) independent village managers, and 3) residents. To recruit residents, a variety of different approaches were used, including distribution of information pack, on-site presentations, free muscle and functional testing, and posters displayed in common areas.

**Results:**

Due to challenges with recruitment, three cohorts were established between February 2014 and April 2015 (14 months). Sixty retirement villages were initially invited, of which 32 declined or did not respond, leaving 28 villages that expressed interest. A total of 3947 individual letters of invitation were subsequently distributed to residents of these villages, from which 517 (13.1%) expressions of interest (EOI) were received. Across three cohorts with different recruitment strategies adopted there were only modest differences in the number of EOI received (10.5 to 15.3%), which suggests that no particular recruitment approach was most effective. Following the initial screening of these residents, 398 (77.0%) participants were deemed eligible to participate, but a final sample of 300 (58.0% of the 517 EOI) consented and was randomized; 7.6% of the 3947 residents invited. Principal reasons for not participating, despite being eligible, were poor health, lack of time and no GP approval.

**Conclusion:**

This study highlights that there are significant challenges associated with recruiting sufficient numbers of older adults from independent living retirement villages into an exercise intervention designed to improve health and well-being.

**Trial registration:**

Australian New Zealand Clinical Trials Registry: ACTRN12613001 161718. Date registered 23rd October 2013.

## Background

Falls among older adults are a serious public health problem, with at least 30% of community-dwelling older adults over the age of 65 years, and 50% over the age of 80 years, falling each year [1, 2]. The consequence of falling among older adults includes injury and fractures, which can result in a loss of independence, reduced quality of life and an upward trajectory in injury-related health care utilization [3, 4]. With the incidence of falls increasing with our aging population, there is currently a concerted effort to implement safe, effective and cost-effective falls prevention programs to a broad range of older adults residing in the community [5]. A healthier, independent older population will reduce the substantial direct and indirect health care costs that are attributable to falls and fractures [6].

In Australia, there has been an increased trajectory in the number of older adults relocating to ‘retirement villages’ or ‘retirement communities’: modes of housing for older adults located in a gated community or complex that often consists of group housing or independent living villas or apartments for adults aged 55 years and over. Currently, Australia has > 2300 retirement villages, of which 184,000 (5.7%) men and women over the age of 55 years reside [7]. By 2025, the number of residents is projected to increase to over 380,000 older adults [7]. These retirement communities are designed to offer older adults a range of health, leisure and support services as well as providing opportunities for increased social interaction and a sense of belonging in a safe and secure environment [8]. In this context, retirement villages represent a captive audience in which to recruit older adults into intervention trials.

Previous research has reported challenges with regard to the recruitment of older adults into fall prevention trials, particularly exercise interventions, as many older adults are often in denial that they are at risk of falling [9, 10]. Randomized controlled trials (RCT) provide the highest level of evidence for health and clinical outcomes; however, the successful recruitment of participants is critical to ensure the external and internal validity of results [11]. One of the major challenges when conducting any RCT in older adults is finding an adequate balance between the recruitment requirements, study timeline and allocated budget [11, 12]. Failing to recruit the required number of eligible participants within these constraints can significantly impact the statistical power of the trial and ultimately the overall findings [11]. Currently, there is limited data on successful recruitment strategies of older adults into fall prevention programs, particularly those residing in independent living retirement villages.

The aim of this report is to describe the strategies, efficacy, pitfalls and successes associated with recruiting 300 older adults residing in retirement communities at increased risk for falling, into an 18-month RCT referred to as the Physical and Cognitive Exercise Intervention Trial (PACE-IT). This is a community-based, cluster RCT, with a 6-month supervised and structured intervention, a 6-month ‘step-down’ maintenance phase and a 6-month follow-up, in which older adults residing in retirement villages, who were at a high risk of falling, were randomly allocated to either an exercise program involving dual-task functional power training (DT-FPT), or a usual care control group. The primary aim of the RCT was to determine whether DT-FPT could reduce the rate of falls in older adults residing in independent living retirement communities [13]. The initial goal was to recruit 280 older adults from a total of 14–16 retirement villages within Melbourne, Victoria over a 12-month period. Here we provide a detailed report of the recruitment approaches and subsequent successes and failures associated with this RCT, in order to inform strategies for future trials aimed at recruiting this population group.

## Methods

### Study overview

A detailed study protocol of the PACE-IT intervention has previously been published [13]. Briefly, this study was an 18-month, community-based, cluster RCT in which older adults residing in retirement villages, who were at a high risk of falling, were randomly allocated to 1) an exercise program involving DT-FPT, or 2) a usual care control group. The intervention was divided into three distinct phases: 6 months of supervised and structured DT-FPT, a 6-month step down maintenance phase and a 6-month follow-up. The primary outcome of this study was the rate of falls over the 6-, 12-, and 18-month study period. Secondary outcome measures assessed at these time points included changes in lower limb functional muscle strength and power, isometric knee extensor, dorsiflexion and grip strength, dynamic balance and reaction time, gait, quality of life, cognitive function and falls related self-efficacy.

Following initial screening and baseline testing, participants were randomised by cluster (retirement village), stratified by village size (< 75 or ≥ 75 residents), to either the DT-FPT program or the usual care group. Participants residing in the villages assigned to the DT-FPT program were asked to attend two supervised exercise sessions per week for 26 weeks followed by a 26-week step-down phase of one supervised session per week. Detailed information about the training program has been reported previously [13]. Briefly, all training was conducted onsite at each retirement village in small groups (8 to 10 per group) and supervised by an accredited exercise physiologist who has completed a University degree or certificate IV fitness trainers who have typical completed an 18-month course that allows them to practice as personal trainers. The 26-week training program was divided into a 2-week familiarisation (orientation) period, followed by three distinct but interrelated 8-week mesocycles, which were designed to be progressively more challenging. Each training session (45–60 min in duration) was divided into four components: 1) a warm-up consisting of rhythmic and range of motion exercises, 2) challenging balance and mobility activities, 3) high-velocity progressive resistance training (HV-PRT), and 4) a cool-down. Dual-task cognitive and motor activities were incorporated simultaneously into the challenging balance, mobility and HV-PRT exercises. All exercise programs were individualized to the participant’s functional ability.

This study was managed by the Institute for Physical Activity and Nutrition (IPAN) at Deakin University, Burwood, Melbourne, Australia, and was funded by a National Health and Medical Research Council (NHMRC) project grant (ID1046267). The study was approved by the Deakin University Human Research Ethics Committee (HREC 2013–051) and was registered with the Australian and New Zealand Clinical Trials Registry (ACTRN12613001161718). Written informed consent was obtained from all participants prior to commencement in the trial.

### Recruitment strategies

This study was conducted in retirement villages within the Melbourne metropolitan region and surrounding areas of regional Victoria, Australia. For this trial, a three-stage recruitment strategy was adopted which included the recruitment of 1) retirement village operators 2) village managers, and 3) residents residing in the retirement villages. These participants were recruited via advertisements placed on community notice boards, and word of mouth from village residents and managers.

#### Recruitment of retirement village operators

The first level of recruitment required identifying all independently living retirement villages within a 125 km radius of Deakin University, Burwood, Victoria, Australia through internet searches, yellow pages, and senior citizen expos. In total, 39 retirement village operational managers were identified, equating to a total of 447 individual villages. The retirement village operational managers oversee all individual village managers and were the first point of contact prior to obtaining permission to contact individual village managers. Retirement village operators with the largest number of independent villages and/or resident numbers and in closest proximity to Deakin University were listed and subsequently contacted in order from the largest to smallest number of village occupants until recruitment saturation. Contact with, and recruitment of, the retirement village operators occurred from November 2013 to December 2014. Initial contact was made first by telephone to determine if the management would be interested in the trial being run within their communities, which was followed up with a face-to-face meeting once interest was confirmed. For each of the three cohorts, the location or distance of each village in relation to metropolitan Melbourne was scattered across the north, south, east and western regions.

#### Recruitment of retirement village managers

The second level of recruitment required the recruitment of the individual retirement villages and their managers. Based on a previous four-month trial from our group [14], we estimated that approximately 14–16 villages would need to be recruited to accommodate the 280 participants needed for this trial (~ 20 participants per village). Managers of the independent villages were initially contacted via email by the operations management to show their support for the trial to be run in the independent villages. Research staff followed up this contact with a village invitation letter sent directly to the manager’s personal work email. A follow-up telephone call was made to all managers following the email to determine the interest and support for the study. Once the managers agreed to participate in the trial, research staff arranged for a study information pack (information letter, EOI form, and a reply-paid envelope) to be delivered to each resident aged 65 years and over residing within the village. In addition, an information session was arranged with the management to be held a week following the mail drop. Posters were also provided to the managers to advertise the trial, and an information session conducted by the research staff was held within the retirement village community centre to promote the study.

#### Recruitment of village residents

The third level of recruitment required the recruitment of the village residents from within the retirement villages. Recruitment of participants was conducted from February 2014 to April 2015. A range of different strategies was used to increase the recruitment of the residents living within each of the villages. First, since it was assumed that many residents within the communities may not have transportation to travel to Deakin University, all aspects of the assessment and intervention were conducted within the retirement community, requiring exercise trainers and researchers to travel to the villages to reduce participant burden. Secondly, to maximize recruitment, the timeline for recruitment for this trial was initially designed to recruit participants over two distinct cohorts in year 1 and 2. For each cohort, all residents residing in the communities received a study information packet delivered to their home inviting them to participate in the trial. Posters were also displayed in all common areas at each community and an on-site information session was provided to educate the residents about ‘healthy ageing’ and the potential health benefits of participating in the trial. The study was not promoted as a falls prevention trial but rather as an intervention designed to improve overall health, mobility and well-being. For each of the two cohorts, residents were asked to indicate their interest in the trial by completing and returning (via a reply-paid envelope) an EOI form to the research staff. Once received, research staff contacted all interested participants from the same village by telephone to determine their eligibility. For a village to be deemed eligible at least six participants had to meet the eligibility criteria for inclusion into the study.

### Eligibility criteria

As reported previously [13], all interested participants were initially assessed for eligibility using a telephone-screening questionnaire. In brief, participants were eligible for the study based on the following criteria: scoring > 3 points on a falls risk algorithm adapted from identified risk factors for falls [15] indicating increased falls risk, able to walk unaided or with minimal assistance for at least 50 m, cognitively intact (score < 2 errors on the Short Portable Mental State questionnaire) [16], and able to speak English. All eligible participants were further screened using the Exercise and Sports Science Australia (ESSA) exercise screening tool to evaluate any contraindicated medical conditions to exercise. Participants answering ‘yes’ to any of these screening questions were required to obtain medical clearance from their local doctor prior to participating in the intervention.

Participants were ineligible for the study based on the following criteria: 1) current or prior participation in a structured progressive resistance training (PRT) program and/or a balance training program more than once per week in the past 3 months, or accumulation of > 150 min of moderate to vigorous physical activity a week; 2) acute or terminal illness likely to compromise exercise participation; 3) unstable or ongoing cardiovascular/respiratory disorders; 4) musculoskeletal or neurological diseases disrupting voluntary movement or that might limit training; 5) upper or lower extremity fracture in the past 6 months, or 6) visual impairment not corrected with glasses.

### Data collection and analysis

Data collection and analyses for this study were conducted at three levels: the village operators, the village managers and the residents. Data were collected regarding the number of operators approached and recruited, village managers recruited, resident EOIs received, and residents screened. Reasons for ineligibility and non-participation in the trial were also recorded at each level of recruitment. Response rates were calculated and reported as the number of operators/villages/residents recruited into the trial divided by the number initially invited. Recruitment yield was calculated as the total number of residents recruited divided by the number of EOIs received.

## Results

### Recruitment timeline

Recruitment of participants for this trial was initially intended to be conducted across two cohorts (12 months apart) each comprising 140 residents (280 in total) from a total of 7–8 retirement villages each year (14–16 in total). However, recruitment was extended to a period of 14 months (February 2014 to April 2015) over three cohorts separated by 6 months due to the lower than expected recruitment rate for the first cohort. Furthermore, the participant recruitment goal was amended from 280 to 310 upon completion of recruitment for cohort 1 to allow for a 10% dropout immediately following baseline testing and prior to the start of the intervention. Overall, 13 participants withdrew from the study following baseline testing and prior to randomization, due to the testing being too difficult or challenging (*n* = 6), health-related reasons (*n* = 6), or a lack of General Practitioner (GP) approval (*n* = 1).

### Response rates, eligibility and reasons for ineligibility or non-participation

**Fig. 1:**
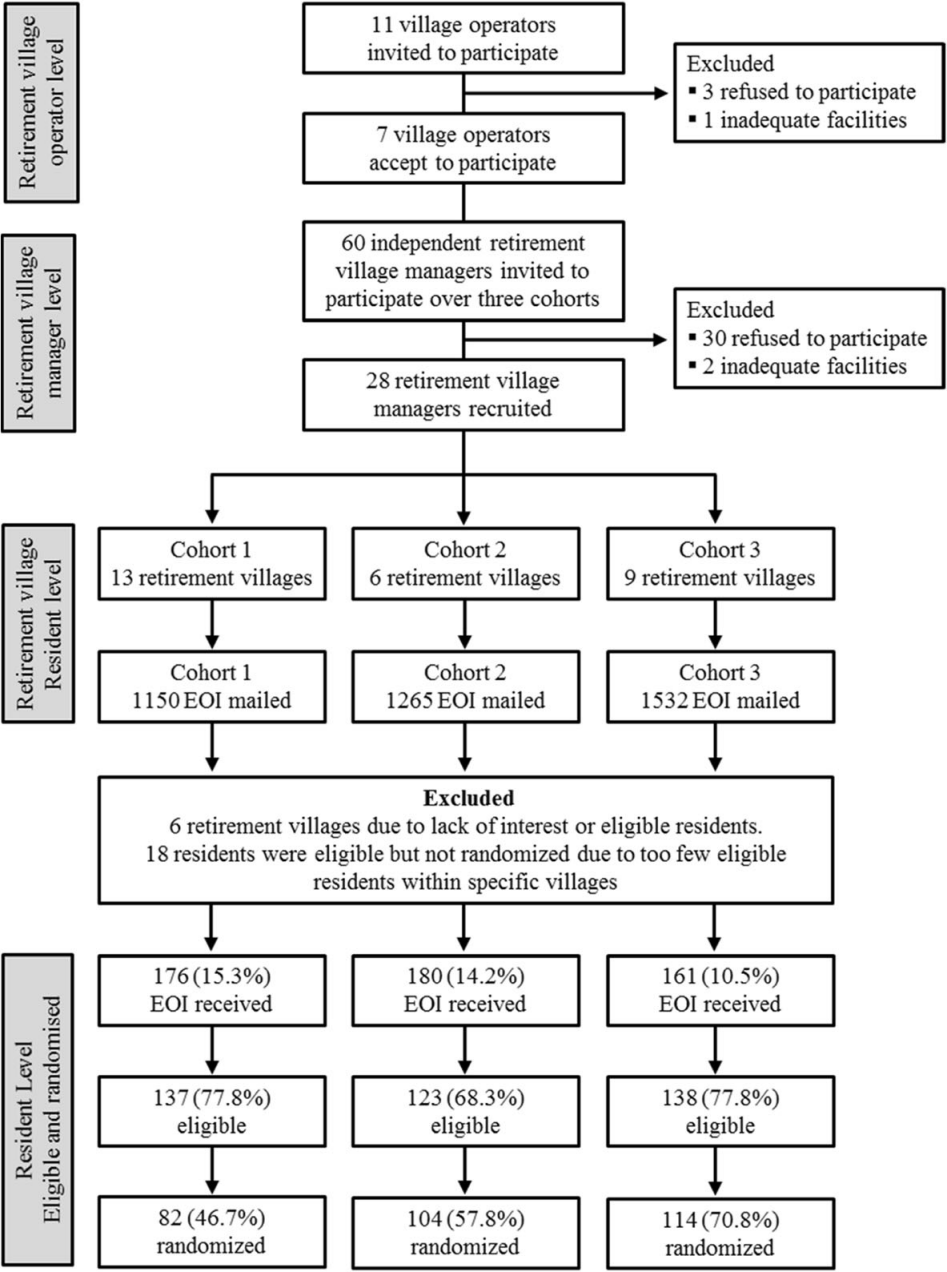
Flow diagram illustrating the recruitment at the operations, management, and resident level within the retirement villages (EOI, expression of interest)

A total of 11 retirement village operators were contacted to participate in the trial, of which three declined and one did not have adequate facilities to implement the intervention. The remaining seven (63.6%) village operators encompassed 60 individual retirement communities. After initial contact with all retirement village managers at each of these communities, a total of 28 (46.7%) were recruited; of the 32 (53.3%) who were not recruited, nine declined and 23 did not respond to the invitation. Overall, 3947 individual letters of invitation were sent to residents across 28 retirement villages, however, only 22 villages were deemed eligible following participant screening as six (21.4%) of the interested villages had an insufficient number of interested and/or eligible residents (Fig. 1).

Of the 3947 letters of invitation which were sent to residents, 517 (13.1%) EOIs were received. Following the screening of these residents, 398 (77.0%) participants were deemed eligible to participate, however, only 300 (58.0%) consented, attended baseline testing and were subsequently randomized. Of the 119 (23.0%) participants deemed ineligible (Table 1), the main reasons were no longer interested or could not be contacted (*n* = 50, 42.0%), being classified as too physically active to meet the eligibility criteria (*n* = 34, 28.6%), and not being classified as at increased risk for falling (*n* = 27, 22.7%). The main reasons for non-participation in the 98 participants that were initially deemed eligible included: exclusion of the retirement village due to low numbers (*n* = 18, 18.4%), poor health (*n* = 11, 11.2%), a lack of time to commit to the study (*n* = 6, 6.1%), and failure to gain GP approval (*n* = 5, 5.1%). Fifty of the 98 participants (51%) provided no reason for non-participation in the trial despite being deemed eligible. Overall 7.6% of the 3947 residents invited to participate were deemed eligible and randomized into the trial.

**Table 1:** Number and proportion of participants deemed eligible and ineligible for the study, including the reasons for ineligibility and non-participation

	Total, n (%)
Total Screened	517 (100%)
Ineligible	119 (23.0%)
Too physically active ^a^	*34 (28.6%)*
Not at a high risk for falls (< 3 risk factors)	*27 (22.7%)*
Poor cognitive function (> 2 errors on the SPMSQ)	*6 (5.0%)*
Not able to walk > 50 m unaided	*1 (0.8%)*
Age < 65 years	*1 (0.8%)*
No longer interested / could not be contacted	*50 (42.0%)*
Potentially eligible	398 (77.0%)
Non-participation after being deemed eligible	98 (8.9%)
No GP approval	*5 (5.1%)*
Poor health	*11 (11.2%)*
Poor health of a spouse	*3 (3.1%)*
Cognitive difficulties following screening	*1 (1.0%)*
Lack of time to commit to the study	*6 (6.1%)*
Not available during the intervention period	*3 (3.1%)*
No longer interested to participate	*1 (1.0%)*
Retirement village had < 6 eligible participants	*18 (18.4%)*
No reason given for non-participation	*50 (51.0%)*
Total Number Enrolled in Study	300

Abbreviations: *SPMSQ* Short Portable Memory State Questionnaire, *GP* general practitioner

^a^ Participants were deemed ineligible if they were meeting or exceeding the Australian National Physical Activity guidelines of > 150 min per week of moderate to vigorous activity, and/or participated in more than one structured resistance (strength) training class or balance class per week

### Recruitment return by recruitment strategies

Over the entire 14-month recruitment period a range of different recruitment strategies were utilized. Cohort 1 was provided with study information packs, which were given to the village managers for distribution and for use at on-site information sessions. However, due to the lower than expected recruitment numbers for this cohort, a number of new recruitment approaches were trialled for cohort 2. This included on-site information sessions, an offer of a free 30-min muscle health and functional assessment, and individually addressed study information packs mailed to the residents via the village managers. Cohort 3 included on-site information sessions and hand delivering of study information packets to residents. Village managers within all three cohorts did not provide permission for the recruitment of participants to be extended to non-village residents that fulfilled the inclusion criteria. As shown in Table 2, the response rate in terms of the EOI received differed between the three cohorts, with both cohort 1 (*n* = 176, 15.3%) and cohort 2 (*n* = 180, 14.2%) yielding a better response compared to cohort 3 (*n* = 161, 10.5%) (chi-squared, *p* < 0.001 and *p* < 0.01 respectively). However, after accounting for the variability between villages for the three cohorts these differences were no longer significant. In contrast, based on the number of EOIs received for each cohort, the recruitment approach adopted during cohort 3 resulted in the greatest number of eligible and subsequently randomized participants (chi-squared, *p* < 0.001, cohort 1: *n* = 82 (46.7%), cohort 2: *n* = 104 (57.8%), cohort 3: *n* = 114 (70.8%). This did not appear to be due to differences in the physical characteristics of participants across cohorts as there were no consistent differences in the physical performance measures (data not shown).

**Table 2:** Numbers of: expressions of interest, the response rate and proportion of residents deemed eligible and ineligible, and those randomized: data presented according to the study cohorts and retirement villages

		Expression of Interest				
	Village	Distributed	Response Rate n (%)	Eligibility Rate ^b^ n (%)	Ineligibility Rate ^b^ n (%)	Randomized ^c^n (%)
Cohort 1 (*n* = 13)	1	70	21 (30.0)	16 (76.2)	5 (23.8)	12 (57.1)
	2	155	35 (22.6)	28 (80.0)	7 (20.0)	11 (39.3)
	3	60	15 (25.0)	14 (93.3)	1 (6.7)	12 (80.0)
	4	65	14 (21.5)	12 (85.7)	2 (14.3)	11 (78.6)
	5	80	12 (15.0)	9 (75.0)	3 (25.0)	7 (58.3)
	6	170	16 (9.4)	12 (75.0)	4 (25.0)	8 (50.0)
	7	126	24 (19.0)	21 (87.5)	3 (12.5)	16 (66.7)
	8	156	14 (9.0)	10 (71.4)	4 (28.6)	5 (35.7)
	9 ^a^	65	5 (7.7)	5 (100)	0 (0)	^a^
	10 ^a^	22	1 (4.5)	0 (0)	1 (100)	^a^
	11 ^a^	56	5 (8.9)	2 (40)	3 (60)	^a^
	12 ^a^	45	9 (20.0)	5 (55.6)	4 (44.4)	^a^
	13 ^a^	80	5 (6.3)	3 (60.0)	2 (40)	^a^
		1150	176 (15.3)	137 (77.8)	39 (22.2)	82 [46.7 (7.1 ^d^)]
Cohort 2 (*n* = 6)	14	262	25 (9.5)	17 (68)	8 (32)	17 (68)
	15	79	12 (15.2)	10 (83.3)	2 (16.7)	10 (83.3)
	16	139	25 (18.0)	18 (72.0)	7 (28)	14 (56.0)
	17	198	35 (17.7)	27 (77.1)	8 (22.9)	23 (65.7)
	18	310	33 (10.6)	24 (72.7)	9 (27.3)	19 (57.6)
	19	277	50 (18.1)	27 (54.0)	23 (46.0)	21 (42.0)
		1265	180 (14.2)	123 (68.3)	57 (31.7)	104 [57.8 (8.2 ^d^)]
Cohort 3 (*n* = 9)	20	120	22 (18.3)	21 (95.5)	1 (4.5)	15 (68.2)
	21	169	13 (7.7)	13 (100)	0 (0)	12 (92.3)
	22	144	19 (13.2)	19 (100)	0 (0)	16 (84.2)
	23	134	14 (10.4)	12 (85.7)	2 (14.3)	8 (57.1)
	24	196	19 (9.7)	17 (89.3)	2 (10.5)	15 (78.9)
	25	154	27 (17.5)	24 (88.9)	3 (11.1)	21 (77.8)
	26	120	16 (13.3)	13 (81.3)	3 (18.7)	12 (75.0)
	27	350	27 (7.7)	16 (59.3)	11 (40.7)	15 (55.6)
	28 ^a^	145	4 (2.8)	3 (75.0)	1 (25.0)	^a^
		1532	161 (10.5)	138 (85.7)	23 (14.2)	114 [70.8 (7.4 ^d^)]
*Total*		3947	517 (13.1)	398 (77.0)	119 (23.0)	300 [58.0 (7.6 ^d^)]

^a^ Retirement villages and participants deemed ineligible following screening due to inadequate participant numbers with a given village (< 6 individuals). Percentage response rates were calculated as a proportion to the number of expressions of interest received

^b^ Eligibility and ineligibility rates were calculated as a proportion of the response rate

^c^ Randomized rates were calculated as the proportion of the response rate

^d^ Randomized rates calculated as the proportion of the number of expression of interest distributed

### Recruitment return by gender and age

**Fig. 2:**
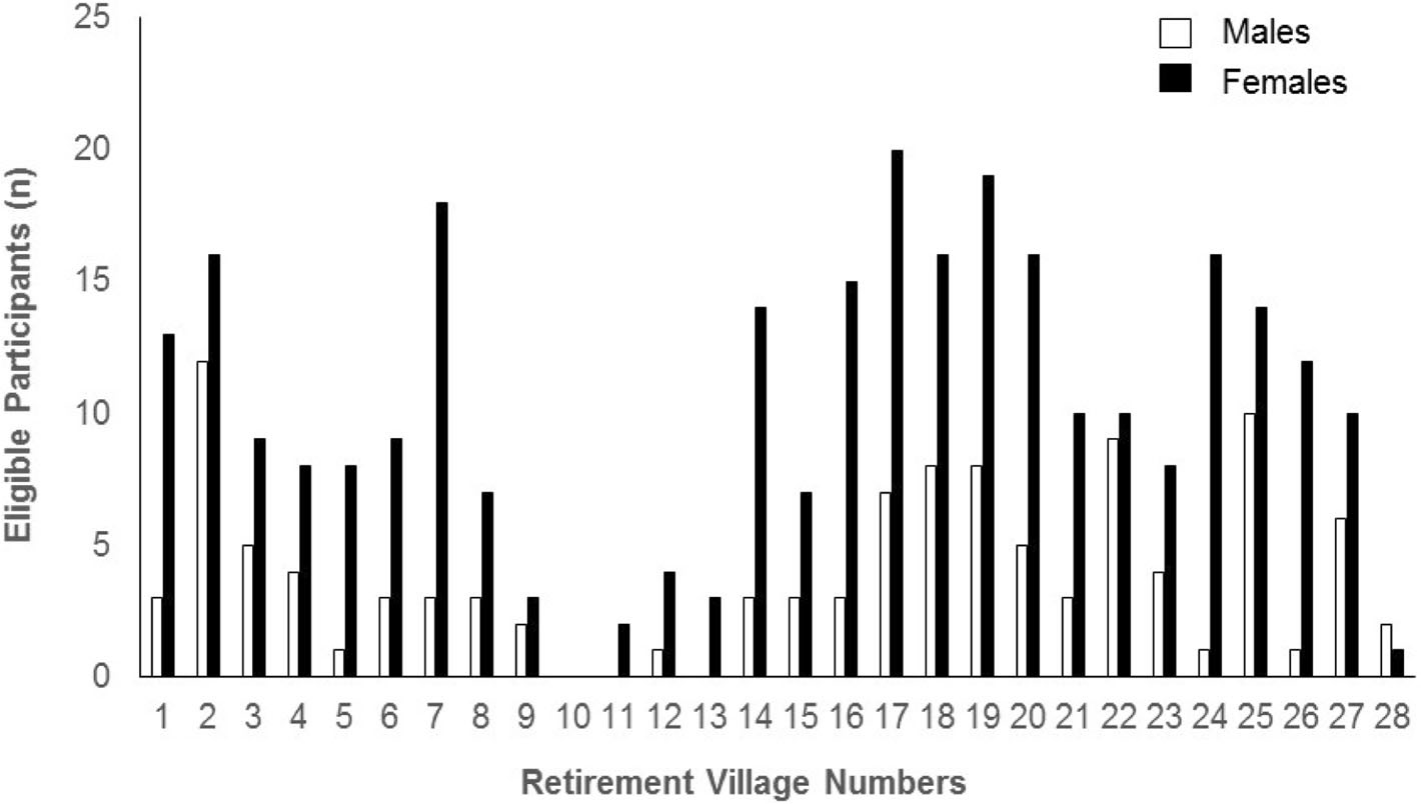
The number of female and male participants (*n* = 398) that were eligible for this trial presented according to the individual retirement villages

Of the 398 participants initially deemed eligible to participate in this trial, the vast majority were female (*n* = 288, 72%). Figure 2 presents the number of males and females recruited at each retirement village over the 14-month period. The average age of the 398 individuals was 77.6 ± 7.2 years; 32% were aged 65–74 years, 46% were aged 75–84 years and 22% were aged > 85 years. When analysing the recruitment return by sex and age of the 300 participants who were randomised, a greater percentage were female (73%) than male, and the mean age for females was on average 1 year younger than males (mean ± SD; female: 77.1 ± 6.1 male: 78.6 ± 6.6 years).

## Discussion

This study highlights that there were significant challenges associated with recruiting operators, managers, and residents of independent living retirement villages into an exercise intervention trial promoted to improve overall health, mobility and well-being. Overall, 64% of village operators, 47% of managers and 13% of residents that were approached expressed an interest in participating in this trial, with 7.6% of residents deemed eligible and randomised to participate in the trial. Because of this low response rate, the recruitment period for this intervention trial had to be extended and a range of different recruitment strategies utilized to reach the desired number of participants for the study. However, adapting the recruitment strategies throughout the study did not significantly increase the response rates in terms of expressions of interest from residents after accounting for variability between the villages for each cohort. In contrast, the recruitment approach adopted during cohort 3 did yield the greatest number of eligible and subsequently randomized participants, which was not explained by differences in physical characteristics.

Well-designed RCTs provide the highest level of evidence for health and clinical outcomes, but finding an adequate balance between the recruitment requirements, study timeline and allocated budget can result in a failure to recruit the required number of eligible participants, thus impacting the statistical power and overall findings of the trial. In Australia, approximately 5.7% of men and women (*n* = 184,000) over the age of 65 years reside in independent living retirement communities [7], and this is projected to increase as our population ages. Thus recruiting people residing in these communities and conducting intervention trials may seem like an ideal opportunity to reach a captive cohort. In our trial, seven of 11 village operational managers agreed to support the study and provide approval for village managers of their 60 retirement villages to be contacted and invited to participate. Of those managers, only approximately half (*n* = 28, 47%) agreed to participate, despite the program being: 1) supported by higher level management; 2) free; 3) managed directly by professional research staff, and 4) with limited time commitment required by the managers. However, consistent with these findings, comparable response rates from managers have been reported in at least two other exercise intervention studies conducted in retirement villages (48–50%) [17, 18]. Collectively, these findings indicate that despite the approval and support from higher-level operational village managers, recruiting residents into such studies requires the support from independent village managers who are ultimately responsible for overseeing the research on-site. In future research, it would be useful to understand barriers and enablers to gain the support of village managers to enhance participation in programs that are beneficial for their residents’ overall health and well-being.

In order to reach our recruitment goal, we utilized a number of different recruitment strategies across each of the three cohorts, some of which were similar to those reported in previous studies conducted in retirement villages [17, 19] and community-dwelling populations [15, 18, 20–22]. These included on-site presentations, advertising posters of the study at each village and delivery of individual information packs to all residents. Based on the similar number of expressions of interest received for each cohort (10.5 to 15.3%), it appears that no one recruitment strategy utilized in our trial was substantially more effective than another. These observations are in contrast to those of previous studies, whereby on-site presentations (66.4%) [18] and individualized mail-outs (70.3%) [15] provided the greatest yield of interest from potential participants. Overall our response rate of 13.0% in terms of the expression of interest and the final recruitment rate of 7.6% is similar to that reported by at least two large-scale clinical trials (5.9 and 11.0%) that aimed to reduce the risk of falls and fracture in older people residing within the community [15, 20]. However, several other similar exercise and falls prevention intervention trials have reported much higher rates of success (12 to 53%) [17, 19, 23]. These marked differences may be predominantly due to our strict inclusion/exclusion criteria as we excluded individuals who were physically active and at low risk for falls. In contrast, other exercise-related intervention trials conducted in similar communities excluded individuals who were unable to walk unaided or had medical complications that precluded their involvement in exercise [17, 19]. However, comparing the recruitment success rates of different trials can be problematic due to differences in inclusion/exclusion criteria, even if the community population (i.e. retirement communities) and study design are comparable.

Understanding the reasons for non-participation in clinical RCTs provides valuable information to guide future recruitment strategies. In our trial, 42% of individuals who initially expressed interest in our study reported that it required ‘too much time commitment’, and thus declined at the screening and informed consent phase. Other studies conducted in retirement villages have reported comparable results in terms of non-participants (39 to 44%) [19, 23]. However, in our trial, we also report a further 19% loss of eligible participants prior to the trial commencement, which was due to some participants not receiving GP approval, poor health and lack of time. While these rates of participant loss prior to trial commencement are similar to that reported in other falls and fracture trials (17–18%) [15, 20], another study involving retirement villages reported a much lower rate of loss (3%) [19]. While this study provided no explanation for the non-participation, it is recommended that all future studies provide detailed information about the reason(s) for non-participation in clinical trials to help guide future recruitment strategies.

### Limitations

There are a number of limitations associated with this study. First, our trial specifically targeted older adults residing in retirement villages who were at an increased risk of falling to participate in a dual-task exercise-training program to reduce falls risk. Therefore, the same experiences may not be evident in other groups residing outside of a purpose built retirement community or clinical population. Second, during recruitment for cohort 1 and 2, we relied on the individual managers rather than research staff to deliver the recruitment information packets to the mailboxes of all residents. Thus, we are unable to comment on the exact method of distribution of the information, nor the reach of that information, and subsequently why some communities had a lower response rate than others. Third, we did not quantify the cost associated with the recruitment of eligible participants into our trial. This would provide valuable information when costing future clinical trials, as failing to recruit the required number of eligible participants within the set budget can significantly impact the overall study findings. Specifically, it would be beneficial to quantify the cost of each individual participant by determining the cost of printing and mailing information, and the staffing costs to deliver recruitment material. Finally, we did not monitor the barriers associated with the recruitment of retirement villages and residents into the trial. However, it is possible that some of the barriers may include a lack of time or even attitudes to ageing and environmental restraints i.e. for managers this may be a lack of understanding of the importance of exercise and for residents there may be a concern about their physical ability to participate and ability to move around the new environment such as the exercise room. Future research should consider addressing barriers associated with recruitment of retirement village managers and residents through qualitative research to investigate possible successful recruitment strategies in this community.

## Conclusions

In conclusion, we have identified that there were significant challenges associated with recruiting operators, managers and residents of retirement villages into a cluster clinical intervention trial designed to evaluate the effectiveness of a dual-task exercise program for preventing falls and improving physical and cognitive function in older people. Although it is common for researchers conducting clinical RCTs to face recruitment difficulties [24], there are currently limited data describing the strategies, efficacy, pitfalls and successes associated with recruiting older adults. In this study, adapting recruitment strategies throughout the study did not significantly increase the response rates in terms of expressions of interest from residents suggesting that no particular recruitment approach was most effective.

Retirement village communities present as a captive, and potentially viable recruitment population for large clinical trials, but we have identified that there are a number of challenges associated in working with this population. Based on the findings from this trial, we have developed a set of five recommendations to optimize recruitment among residents of retirement villages:

1. Given the complexity of recruiting residents within retirement villages, researchers should ensure that adequate time is available to establish rapport with operators, managers and residents, and not underestimate the time frame needed to gain access to the communities.
2. It is important to gain approval and support from the higher level operational managers when planning to conduct research studies in retirement communities, but from a practical perspective, it is the independent village managers who are responsible for supporting and promoting the research on-site and so it is essential to gain their support.
3. Researchers must consider the time commitment of managers and insist on the delivery of advertisements and information packets to all residents by the research staff to ensure all residents receive trial material.
4. Recruitment strategies need to be continually reviewed, developed and refined to maximize the involvement of the retirement village residents into research trials while still protecting their right to refuse.
5. When working in retirement villages researchers must be prepared for an approximate 40% non-participation rate (from those who initially express an interest in the study), and a further ~ 20% loss of eligible participants prior to the study commencement.

### Trial status

Recruitment and data collection for this trial is complete and data analysis has commenced.

## Abbreviations

C: Cohort
DT-FPT: Dual-Task Functional Power Training
EOI: Expression of Interest
ESSA: Exercise and Sports Science Australia
IPAN: Institute for Physical Activity and Nutrition
NHMRC: National Health and Medical Research Council
PACE-IT: Physical and Cognitive Exercise Intervention Trial
PRT: Progressive Resistance Training
RCT: Randomized Controlled Trial
